# Central autonomic network dysfunction in Stroke-Heart Syndrome: mechanistic roles of the insula and limbic system

**DOI:** 10.3389/fnins.2026.1690302

**Published:** 2026-02-18

**Authors:** Hongxin Li, Wang Guo, Qiwen Nie, Zhihao Wang, Hongyu Li

**Affiliations:** 1 Department of Rehabilitation Medicine, Heilongjiang University of Chinese Medicine, Harbin, China; 2 Department of Neurological Rehabilitation, The Second Affiliated Hospital of Heilongjiang University of Chinese Medicine, Harbin, China

**Keywords:** autonomic system dysfunction, central autonomic network, insular cortex, limbic system, Stroke-Heart Syndrome

## Abstract

Stroke-Heart Syndrome describes cardiac dysfunction following acute cerebrovascular events, with injury to the central autonomic network being a key pathological mechanism. The insular cortex and limbic system act as central hubs for neuro-cardiac regulation, integrating autonomic, neuroendocrine, and immune signals. This review summarizes the structural and functional organization of the central autonomic network, emphasizing the insula’s subregional specialization and its hemispheric bias in autonomic regulation. Generally, the right insula is more often linked to sympathetic predominance and the left to parasympathetic modulation, though this pattern is not absolute but rather contingent on subregional and methodological factors. Clinical and experimental evidence links lesions in the insula and limbic system to arrhythmias, QT interval prolongation, and myocardial injury through autonomic imbalance. Advances in neuroimaging, such as functional magnetic resonance imaging and diffusion tensor imaging, provide novel biomarkers for early cardiac risk stratification after stroke. Furthermore, emerging interventions including heart rate variability biofeedback and non-invasive vagus nerve stimulation show therapeutic potential by targeting these central circuits. Elucidating the mechanisms of central autonomic network injury, particularly involving the insula and limbic system, is essential for improving risk assessment and developing targeted therapies for Stroke-Heart Syndrome.

## 1 Introduction

**Figure 1:**
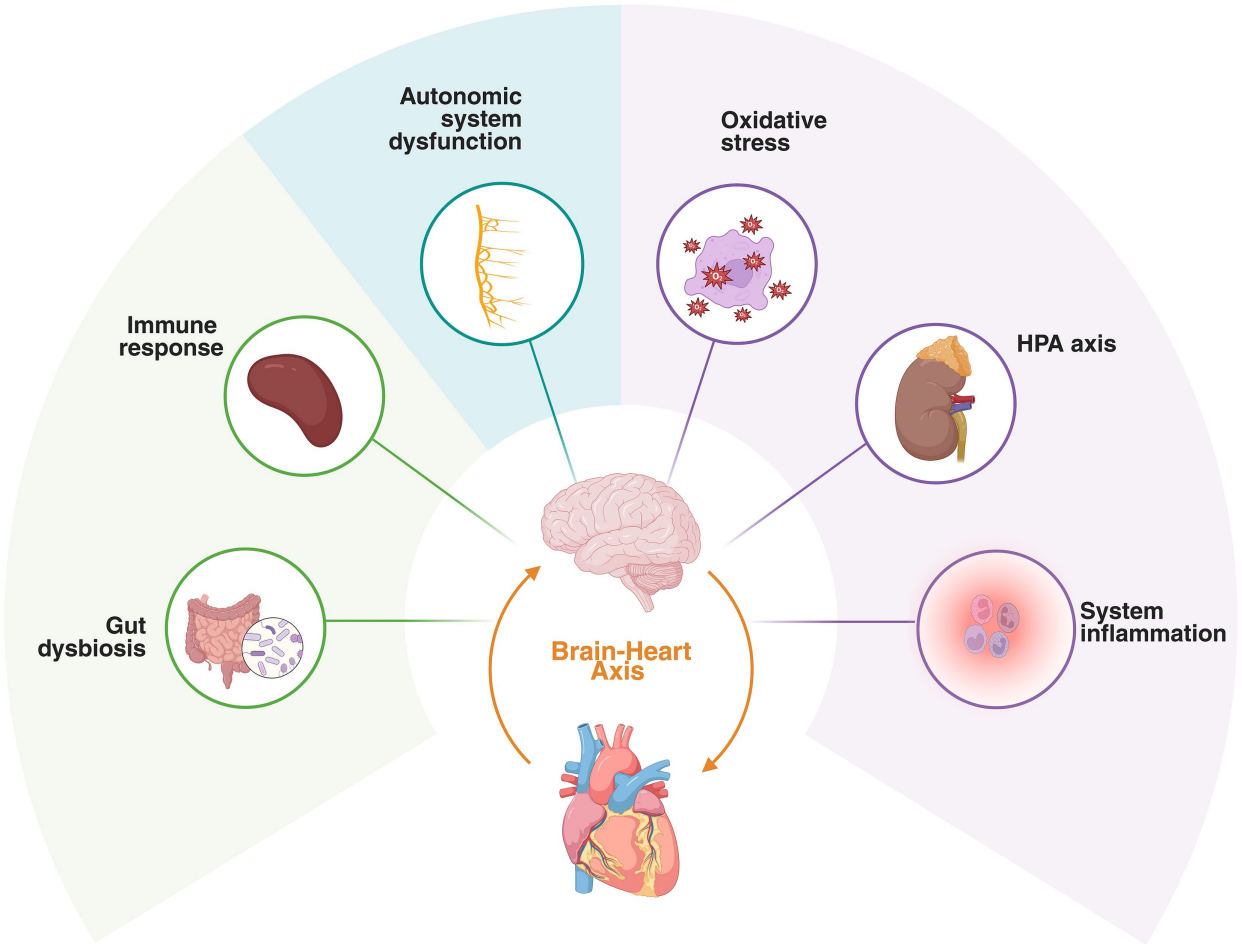
Pathophysiological mechanisms of Stroke-Heart Syndrome. Stroke can induce autonomic dysfunction, intestinal microbiota dysbiosis, oxidative stress, hypothalamic–pituitary–adrenal axis activation, inflammation, and immune responses. These mechanisms exhibit intricate interactions, potentially culminating in cardiac injury. Created in https://BioRender.com.

Stroke remains one of the leading causes of disability and death worldwide (Martin et al., 2025). Cardiac complications are frequent within the first month after stroke and strongly predict worse long-term outcomes, including higher all-cause mortality, recurrent stroke, and major adverse cardiovascular events (Buckley et al., 2022; Buckley et al., 2024; Sposato et al., 2020b). These complications now represent the second leading cause of death in the acute phase, and the widespread adoption of mechanical thrombectomy may further increase this burden (Laugesen et al., 2025; Prosser et al., 2007). Together, these observations have led to the definition of Stroke-Heart Syndrome (SHS), a unifying framework describing secondary cardiac injury following stroke (Scheitz et al., 2018). SHS encompasses a broad clinical spectrum of cardiovascular changes including (1) acute myocardial injury (as evidenced by acute elevation of high-sensitivity cardiac troponin [hs-cTn]), (2) acute coronary syndromes (ACS; including type 1 and type 2 myocardial infarction [MI]), (3) systolic and diastolic LV dysfunction including Takotsubo syndrome (TTS), (4) cardiac arrhythmias including atrial fibrillation (AF) and relevant ECG changes, and (5) neurogenic sudden cardiac death (Fan et al., 2024). Its underlying pathobiology extends beyond conventional cardiovascular risk factors, involving disruption of the brain–heart axis (BHA)—including autonomic dysregulation, neuroendocrine stress activation, oxidative stress, gut dysbiosis, and immune–inflammatory cascades (Sposato et al., 2020a), as illustrated in Figure 1.

Central autonomic control is organized within a distributed Central Autonomic Network (CAN) that links cortical, limbic, hypothalamic, brainstem, and spinal nodes (Chouchou et al., 2025). Neuroimaging and network-based lesion mapping have shown that damage involving the insula and limbic hubs closely corresponds to autonomic dysregulation and cardiac dysfunction, underscoring a systems-level rather than focal locus of control (Reisert et al., 2021; Ruffle et al., 2021; Templin et al., 2019). However, current research on SHS has focused predominantly on peripheral and inflammatory mechanisms, while central network contributions remain comparatively underexplored (Liang et al., 2024; Simats et al., 2024; Yan et al., 2020). This review integrates neuroimaging, experimental, and clinical evidence to elucidate how the insula and limbic system within the CAN contribute to post-stroke autonomic imbalance and subsequent cardiac dysfunction. We further highlight mechanistic pathways linking central circuitry to myocardial injury and outline emerging neurocentric biomarkers and targeted interventions that may enable earlier risk stratification and precision therapy for SHS.

### 1.1 Operationalizing Stroke-Heart Syndrome: diagnosis and differential challenges

Distinguishing SHS from ACS is challenging due to prevalent silent coronary disease and masked ischemic symptoms (Scheitz et al., 2022). hs-cTn elevation occurs in ~50% of acute stroke patients but is non-specific, potentially reflecting Type 1 MI, Type 2 MI (supply–demand mismatch), neurogenic injury, or renal dysfunction (Ahn et al., 2023; Scheitz et al., 2021). Crucially, a prospective trial found that among stroke patients with elevated hs-cTn, only ~20% had Type 1 MI. While dynamic changes failed to identify thrombotic occlusion, baseline values >5-fold the upper limit of normal (ULN) were strongly predictive (Nolte et al., 2024).

Operationally, SHS represents acute neurogenic cardiac dysfunction sans coronary thrombosis, though “hybrid scenarios” where stroke stress unmasks underlying CAD are common (Hinterdobler et al., 2021; Roth et al., 2018). To reduce neurogenic over-attribution, we advocate a pragmatic diagnostic algorithm: (1) Biomarker Interpretation: use serial hs-cTn to confirm acute injury, but rely on high absolute baseline values (>5 × ULN) rather than dynamics to flag Type 1 MI; (2) Mechanism Phenotyping: rigorously screen for Type 2 MI triggers like sustained tachyarrhythmia or hypertensive crisis; and (3) Imaging Patterns: prioritize echocardiography to differentiate the diffuse repolarization changes (e.g., widespread T-wave inversion) and non-coronary wall motion abnormalities (e.g., apical ballooning) typical of SHS from the vascular-territory restricted deficits of ACS. Invasive angiography should be reserved for patients with high probability of Type 1 MI.

## 2 The central autonomic network: hierarchical structure and systemic integration

### 2.1 Hierarchical organization of the central autonomic network

The CAN comprises a hierarchical network of structures distributed throughout the central nervous system, extending from higher-order cortical regions such as the cerebral cortex and basal ganglia, descending through the midbrain and brainstem, and ultimately reaching the spinal cord (Benarroch, 1993). The principal nodes of the CAN include the insular cortex (IC), anterior cingulate cortex (ACC), prefrontal cortex (PFC), amygdala, hypothalamus, and the medullary nucleus tractus solitarius (NTS) (Ferraro et al., 2022). These regions collectively orchestrate the dynamic regulation of sympathetic and parasympathetic outflow and integrate autonomic control with complex behavioral, emotional, and homeostatic processes (LeDoux, 2007; Shields, 1993). Recent human single-neuron recordings have demonstrated that thalamic neurons encode both cardiac and respiratory rhythms, while animal and human circuit-mapping studies identify the medial and intralaminar thalamic nuclei as key relays transmitting visceral and nociceptive afferents to the ACC, insula, and brainstem—together underscoring the integrative role of the thalamus in visceral–autonomic processing (Chang et al., 2024; De Falco et al., 2024; Kumar et al., 2023). Although the insula, ACC, amygdala, and PFC form the canonical core of the CAN, recent evidence from direct cortical stimulation in humans suggests a more complex and hierarchically organized network. A large-scale stereoelectroencephalography study revealed that, in addition to a core autonomic network producing robust cardiac responses, a broader distributed network—including sensorimotor, premotor, and lateral temporal cortices—also contributes to autonomic modulation, albeit to a lesser degree (Chouchou et al., 2025). This expanded model broadens the traditional definition of the CAN, highlighting the continuous contribution of neocortical regions to autonomic adjustments during motor, sensory, and cognitive activity. Complementing these human findings, transneuronal tracing in nonhuman primates has delineated direct cortical projections influencing sympathetic outflow to the adrenal medulla, originating from motor, premotor, cingulate, and medial prefrontal cortices (Dum et al., 2016). These studies provide anatomical confirmation of corticogenic pathways mediating autonomic control.

**Figure 2:**
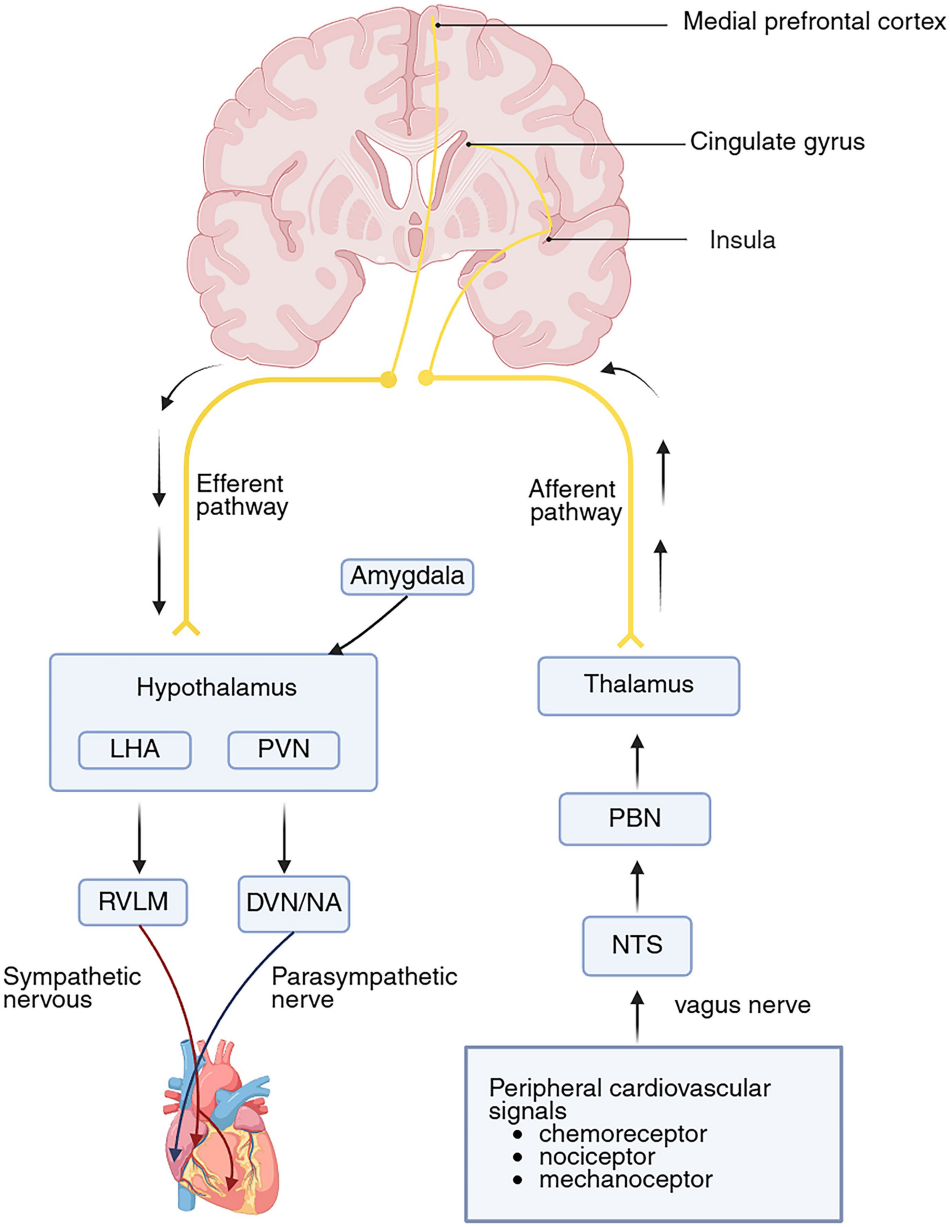
Neural pathways of the brain–heart axis. Ascending cardiovascular signals are relayed through brainstem nuclei (NTS, PBN) and the thalamus to the insular cortex for integration. Descending cortical modulation from the mPFC and ACC converges on the hypothalamus, which integrates emotional input from the amygdala and directs dual autonomic outflow via NA/DVN (parasympathetic) and RVLM (sympathetic) to the heart. The anterior insula translates interoceptive states into autonomic responses, thereby closing the regulatory feedback loop. NTS, nucleus tractus solitarius; PBN, parabrachial nucleus; RVLM, rostral ventrolateral medulla; PVN, paraventricular nucleus; LHA, lateral hypothal area; NA, nucleus ambiguus; DVN, dorsal vagus nucleus. Created in https://BioRender.com.

Together, the neural circuits constituting the brain–heart axis illustrate explicit bidirectionality. The “top-down” efferent pathway originates in the medial prefrontal cortex and insula, projecting to cardiomyocytes and coronary endothelium to modulate cardiac function (Hafez and Chang, 2025). Conversely, the “bottom-up” afferent pathway arises from peripheral sensory receptors and ascends via the brainstem and thalamus to the insular cortex, where higher-order integration of autonomic and interoceptive signals occurs, as illustrated in Figure 2.

### 2.2 Functional integration: neurocardiac, neuroendocrine, and immune dysregulation

#### 2.2.1 Neurocardiac modulation: the primary efferent pathway

The central nervous system regulates autonomic output with remarkable spatiotemporal precision, modulating cardiovascular function in a task-dependent manner via the CAN (Benarroch, 1993; Lohman et al., 2024; Valenza, 2023). Descending efferent pathways transmit CAN-derived signals to the sympathetic and parasympathetic branches of the autonomic nervous system (ANS), exerting direct control over heart rate, contractility, and vascular tone (Beissner et al., 2013; Valenza et al., 2024). Within the brainstem, nuclei in the medulla and pons coordinate circulatory, respiratory, and visceral functions through descending projections to spinal autonomic centers (Trevizan-Baú et al., 2021).

The rostral ventrolateral medulla (RVLM) acts as a critical sympathetic efferent hub, regulating acute blood pressure and hemodynamics by increasing myocardial contractility, accelerating heart rate, and promoting peripheral vasoconstriction (Wenker et al., 2017). Conversely, the nucleus ambiguus (NA) and dorsal motor nucleus of the vagus (DVN) constitute the primary sources of cardiac parasympathetic outflow, as established in classic animal studies (Davis et al., 2004). Both receive afferent input from the NTS and are modulated by descending projections from the hypothalamus (Gasparini et al., 2020). Tracing studies further demonstrate that the paraventricular nucleus (PVN) modulates cardiac vagal outflow via direct connections to the dorsal vagal complex and polysynaptic pathways engaging the NA, thereby coordinating rapid (NA) and tonic (DVN) vagal control (Geerling et al., 2010; Ulrich-Lai and Herman, 2009).

The upper brainstem serves as an integration hub for autonomic and respiratory networks (Farmer et al., 2016). Experimental data demonstrate that respiratory pattern-generating circuits within the medulla and pons gate vagal (NA/DVN) and sympathetic (RVLM) outflows, producing respiratory sinus arrhythmia and respiration-locked oscillations in sympathetic activity (Guyenet and Stornetta, 2022; Hirsch and Bishop, 1981). Thus, breathing frequency and tidal volume directly shape heart rate and blood pressure variability (Trevizan-Baú et al., 2021).

At the forebrain level, the prefronto-limbic circuitry—including the insula, ACC, amygdala, and hypothalamus—translates sensory and emotional stimuli into autonomic responses (Lamotte et al., 2021; Wehrwein et al., 2016). Through relays in the hypothalamus and periaqueductal gray, these cortical hubs influence brainstem respiratory pattern generators, enabling top-down coupling between respiration and autonomic state within the CAN (Krohn et al., 2023; Schottelkotte and Crone, 2022). The ACC plays a key coordinating role, balancing sympathetic and parasympathetic output through its connections with the insula, prefrontal cortex, amygdala, hypothalamus, and brainstem (Lamotte et al., 2021). Distinct regions within the CAN demonstrate robust correlations with autonomic indices such as heart rate (HR), blood pressure (BP), and heart rate variability (HRV) (Matusik et al., 2023).

Clinical studies show that post-stroke abnormalities in HRV reflect disruptions of resting-state brain networks rather than isolated focal lesions (Dimova et al., 2024). Independent-component neuroimaging analyses reveal that reductions in low-frequency and overall HRV are predominantly right-hemispheric, attributable to dysfunction within limbic and ventral attention networks (Dimova et al., 2024). Functional connectivity within the CAN also differs across individuals with varying resting heart rates (de la Cruz et al., 2019). Moreover, effective connectivity from the right amygdala to the ACC and bilateral ventrolateral prefrontal cortex negatively correlates with HRV and positively with HR, suggesting that heightened amygdalo-cortical drive may bias the system toward sympathetic dominance (Ma et al., 2024). In line with these findings, inhibition of lateral tegmental catecholaminergic cell groups produces marked reductions in BP, HR, and sympathetic outflow (Geraldes et al., 2023).

#### 2.2.2 Integrated stress responses: the convergence of autonomic, endocrine, and immune pathways

Following stroke, dysregulation of the CAN initiates a complex neuroendocrine–immune cascade in which autonomic imbalance acts both as a trigger and as a target of secondary injury (Cui et al., 2021). The hypothalamus, serving as the central integrator of limbic and cortical inputs, coordinates this response by co-activating the sympathetic–adrenal–medullary (SAM) system and the hypothalamic–pituitary–adrenal (HPA) axis (Schaeuble and Myers, 2022). Excessive activation of the HPA axis elevates cortisol levels and stimulates the renin–angiotensin–aldosterone system (RAAS), thereby intensifying hemodynamic load and vascular stress (Mohammed et al., 2020; Welcome and Mastorakis, 2020).

This neuroendocrine surge operates in concert with an inflammatory response rather than in isolation. Stroke-induced release of damage-associated molecular patterns (DAMPs) provokes neuroinflammation within the brain parenchyma, characterized by the production of pro-inflammatory cytokines and glial activation (Zarate et al., 2024). Such central inflammation further heightens sympathetic excitability, forming a self-reinforcing feedback loop wherein inflammation and autonomic dysfunction perpetuate each other (Badoer, 2022).

The catecholamine surge generated via the SAM pathway extends beyond the central nervous system, targeting peripheral immune organs such as the spleen and bone marrow to initiate systemic inflammatory activation and leukocyte mobilization (Felten et al., 1985; Katayama et al., 2006). This peripheral immune activation promotes recruitment of inflammatory cells to the myocardium and vascular endothelium, driving maladaptive cardiac remodeling and peripheral vascular injury (Chen H. R. et al., 2022; Hu et al., 2023; Lin et al., 2024; Singh et al., 2019). In essence, sympathetic hyperactivity emerging from the CAN simultaneously activates endocrine and immune pathways, which, in turn, feedback to exacerbate central dysautonomia. This reciprocal cross-talk establishes a fully integrated, self-perpetuating network that underlies the pathophysiology of SHS.

## 3 The insular cortex: a nexus for neurocardiac control

### 3.1 Structural and functional topography of the insula

#### 3.1.1 Subregional specialization: anterior–posterior and dorsoventral gradients

The insular cortex, characterized by its complex cytoarchitecture and extensive interconnections, functions as a pivotal cortical hub that integrates interoceptive awareness, multimodal sensory processing, autonomic regulation, and socio-emotional cognition (Benarroch, 2019). Based on its distinct laminar architecture—comprising granular, dysgranular, and agranular territories—and divergent connectivity profiles, the insula can be anatomically and functionally divided into anterior, middle, and posterior subregions (Gogolla, 2017). The posterior insula primarily receives afferent sensory input related to pain, temperature, visceral, and vestibular sensations (Nguyen et al., 2016). These inputs are integrated within the middle insula and relayed to the anterior insula for higher-order processing and coupling with circuits that mediate cognitive and emotional regulation (Heydrich and Blanke, 2013; Sridharan et al., 2008). This posterior-to-anterior gradient thus reflects a progressive transformation from primary interoceptive representation to abstract emotional and cognitive integration.

Functional specialization also follows a dorsoventral gradient: the dorsal insula is predominantly engaged in sensorimotor control, while the ventral insula is preferentially involved in visceral, affective, and autonomic functions (Evrard, 2019; Krockenberger et al., 2023). Within this framework, visceromotor, emotional, and self-referential processes are represented ventrally, whereas skeletomotor and non-self-referential behaviors are mapped dorsally. Moreover, motor representations follow a posterior-to-anterior somatotopic gradient—from spinal (foot/hand) to cranial (head/face) domains (Simone et al., 2025).

The insula, through its intrinsic microcircuitry and extensive afferent/efferent connections, plays a critical role in cardiac interoception—the conscious perception of heartbeat signals—which underlies self-awareness and emotional salience (Keller et al., 2020). The fidelity of this interoceptive perception depends on the structural and functional integrity of insular networks (Fermin et al., 2023). Dynamic interactions between somatosensory input and cardiac activity further highlight the insula’s role as a mediator of brain–heart coupling (Al et al., 2020). Heightened interoceptive awareness of cardiac activity preferentially recruits the right anterior insula, enhancing empathic sensitivity and modulating arousal and emotional states (Di Bello et al., 2023; Haruki and Ogawa, 2021; Holtmann et al., 2022).

Emerging evidence indicates that the dorsolateral anterior insula maintains a bidirectional regulatory relationship with the ANS: it receives visceral feedback (e.g., heart rate, blood pressure) and, via reciprocal communication, can either stabilize sensory homeostasis or amplify interoceptive salience (Cechetto, 2014; Craig, 2009b; Uddin et al., 2017). Thus, the insula acts as an integrative interface linking somatosensory, visceral, and emotional domains—supporting perceptual awareness, social cognition, and goal-directed decision-making (Zaki et al., 2012).

Acute or chronic insults to the insula—resulting from ischemic stroke, epileptiform activity, or neurodegenerative disease—can disrupt this intricate circuitry, producing a broad spectrum of sensory, autonomic, motor, cognitive, and behavioral disturbances (Belem-Filho et al., 2025; Duong et al., 2025; Lotze, 2024; Tisserand et al., 2024; Wang et al., 2025).

#### 3.1.2 Insula-limbic system connectivity

The insular cortex regulates homeostatic balance, interoceptive processing, and emotional awareness through its extensive structural and functional connectivity with diverse cortical and subcortical regions (Craig, 2009a; Ibañez et al., 2010; McDonald, 1998; Wang et al., 2023). Serving as a multimodal integrative hub, the insula links emotional, interoceptive, and homeostatic domains within a unified network (Damasio et al., 2000). Its connectivity follows a tripartite organizational principle encompassing cognition, emotion, and interoception (Chang et al., 2013; Deen et al., 2011; Uddin et al., 2014): the dorsal anterior insula interacts with the ACC and frontal cortices to mediate higher-order cognitive control; the ventral anterior insula connects with limbic structures to process affective states; and the posterior insula integrates somatosensory and visceral inputs with motor output.

Dense reciprocal projections link the insula and the amygdala complex, forming a core pathway for emotional–autonomic integration (Augustine, 1996; Cloutman et al., 2012; Mufson et al., 1981). Anatomically, the posterior insula projects mainly to the dorsal lateral and central nuclei of the amygdala, while the anterior insula connects with the anterior, medial, cortical, and basolateral nuclei (Shi and Cassell, 1998). Advanced diffusion tractography and fiber dissection studies further reveal a direct amygdalo–insular pathway—the extreme capsule amygdalo-insular bundle—that bridges the insula, amygdala, and medial temporal lobe, forming a rapid conduit for interoceptive–emotional signaling (Nachtergaele et al., 2020). Following stroke, disruption of this amygdalo-insular connectivity, together with altered neuropeptide signaling and sympathetic overactivation, can precipitate excessive norepinephrine release, contributing to neurogenic cardiac dysfunction (Cheung and Cechetto, 1995).

The insula also maintains bidirectional connections with the cingulate cortex, which are essential for integrating visceral sensory and motor information and for sustaining the autonomic salience network (Cormie et al., 2023). The anterior insula primarily connects with the ACC, whereas the posterior insula preferentially interacts with the posterior cingulate cortex (Cauda et al., 2011). Distinct cingulate subregions can differentially modulate the locus coeruleus–norepinephrine system, suggesting a graded organization of emotional–autonomic control (Salvi et al., 2025). Interoceptive and visceral signals reaching the posterior insula are relayed forward to the anterior insula for multimodal integration and subsequently transmitted to the anterior mid-cingulate cortex, enabling dynamic autonomic regulation (Menon and Uddin, 2010).

Von Economo neurons (VENs) within the anterior insula and ACC—specialized for rapid long-range signaling—project descending fibers that modulate autonomic output via the periaqueductal gray and paracentral nucleus, facilitating adaptive cardiovascular and interoceptive responses to prediction errors (Barrett and Simmons, 2015; Seth and Critchley, 2013). These unique neurons may underlie the rapid integration of emotional salience and autonomic state required for survival behaviors.

Collectively, these anatomical and functional pathways establish a robust neurobiological substrate for the tight coupling between the insula, limbic system, and autonomic regulation. Importantly, while the insula–limbic hub serves as the core of the CAN, it operates within a broader, hierarchically distributed system that includes sensorimotor, cognitive, and associative cortices. This extended network likely sustains baseline autonomic tone during everyday behavior, whereas the insula–limbic core is preferentially recruited for salient or stress-related autonomic responses (Chouchou et al., 2025).

### 3.2 Insular lesions and cardiac dysautonomia

#### 3.2.1 Hemispheric specialization: from clinical correlates to causal mechanisms

Investigations into insular stroke and autonomic lateralization reveal a hemispheric bias rather than a strict dominance pattern. This lateral bias is architecturally supported by the asymmetric distribution of VENs within key CAN nodes like the insula and ACC (Allman et al., 2011). Postmortem and MRI morphometric studies confirm that the right hemisphere contains a significantly higher density of VENs than the left (Shaw et al., 2009; Watkins et al., 2001). While these neurons integrate signals to modulate autonomic tone, they likely create a structural basis for “flexible, context-dependent” lateralization rather than fixed functional dominance (Jacot-Descombes et al., 2020).

In this framework, it is critical to distinguish between associative human data and causal animal evidence. At the clinical level, evidence is primarily associative: human correlation studies generally link the right anterior insula with sympathetic predominance and the left with parasympathetic modulation (Holtmann et al., 2022; Keller et al., 2020). These observations are supported by intraoperative electrical stimulation, where right insular activation typically increases blood pressure and heart rate, whereas left-sided stimulation induces hypotensive or bradycardic effects (Sanchez-Larsen et al., 2024). Conversely, animal models provide critical causal support for this asymmetry. Targeted microstimulation and glutamatergic tracing in rodents identify the middle-posterior insula as a primary locus for sympathetic excitation via projections to the dorsomedial hypothalamus (Marins et al., 2021). These experimental findings define the mechanistic boundaries of the CAN, though they represent focal responses compared to the system-wide disruption seen in stroke.

Nevertheless, the evidence for absolute lateralization is not entirely consistent across clinical cohorts. Several studies report bilateral involvement or even reversed autonomic responses, suggesting that insular control operates via dynamically coupled inter-hemispheric networks (Krause et al., 2017; Sanchez-Larsen et al., 2021). We weigh this heterogeneity as a reflection of several systematic methodological and biological confounds: (1) Lesion Topology and Network Connectivity: Unlike focal animal lesions, ischemic stroke often affects white matter fiber tracts (Zhang et al., 2019), leading to distal network dysfunction (diaschisis) that obscures primary hemispheric bias; (2) Temporal Fluctuations: Autonomic tone is highly dynamic post-stroke; assessment timing (e.g., hyper-acute within 24 h vs. subacute phase) significantly impacts whether a unilateral or bilateral phenotype is observed. (3) Variations in HRV methodology—such as reliance on frequency-domain versus noise-resilient time-domain indices (e.g., RMSSD)—often lead to divergent results across centers. Overall, current data support a flexible model where the right insula favors sympathetic and the left supports parasympathetic modulation, yet these effects are probabilistic and contingent on network integrity and subregional specialization.

#### 3.2.2 Clinical manifestations and the biomarker gap

A strong clinical relationship has been established between acute insular stroke and life-threatening disturbances in cardiac autonomic regulation. Extensive associative studies indicate that lesions involving the insular cortex are frequently linked to a spectrum of adverse cardiac outcomes, including sinus tachycardia (Christensen et al., 2005), new-onset AF (Kim et al., 2024), increased blood pressure (Sanchez-Larsen et al., 2024), reduced left ventricular ejection fraction (LVEF) (Winder et al., 2023), decreased HRV (Constantinescu et al., 2019; Kitamura et al., 2018), electrocardiographic abnormalities (Abboud et al., 2006) and higher mortality risk (Hanne et al., 2017). While right-sided involvement is often predominantly cited, clinical manifestations appear probabilistic rather than absolute. Comparable autonomic alterations have been observed following left-sided or bilateral injury, suggesting that cardiac sequelae are shaped by overall network-level disruption and pre-stroke cardiac reserve (Laowattana et al., 2006; Scheitz et al., 2015).

The use of high-sensitivity cardiac troponin (hs-cTn) has provided a highly specific, albeit complex, biomarker for myocardial injury within this syndrome. Elevated hs-cTn occurs in approximately 50% of acute stroke patients and is independently associated with worse long-term mortality and heart failure (Ahn et al., 2017; Ahn et al., 2023). However, the pathophysiological basis for this elevation remains a subject of intense debate. We weigh the inconsistencies observed in lesion-biomarker mapping as reflections of the multifactorial nature of SHS. While voxel-based mapping frequently associates right dorsal anterior insular damage with elevated hs-cTn (Song et al., 2008), other cohorts implicate non-insular or diffuse lesions (Min et al., 2022). These discrepancies highlight several systematic confounds: (1) Stroke Severity: Extensive infarctions may trigger a generalized catecholamine storm, masking the specific influence of individual CAN nodes; (2) Biomarker Kinetics: Differences in peak hs-cTn sampling times often capture different phases of neurogenic injury; and (3) Comorbid Disease: Pre-existing coronary disease can be unmasked by the sympathetic surge of a stroke.

Cortical–autonomic disintegration is further mirrored by characteristic electrocardiographic repolarization signatures, most notably prolonged QTc, increased QT dispersion, and dynamic T-wave inversion. These changes closely resemble the patterns seen in Takotsubo cardiomyopathy and subarachnoid hemorrhage (Ahn et al., 2021; Kuris et al., 2024; Poudel et al., 2023; Wang et al., 2024). These repolarization abnormalities typically peak within the first few days of stroke, fluctuate in tandem with sympathetic-vagal tone, and correlate strongly with reduced HRV (Tang et al., 2020). From a translational perspective, the clinical value of SHS markers lies in their integration: combining standardized QTc dispersion and HRV indices with serial hs-cTn monitoring enables more precise risk stratification for malignant arrhythmias and neurogenic stress cardiomyopathy (Zhang X. et al., 2024). This multimodal diagnostic approach bridges the gap between the mechanistically inferential imaging markers and actionable clinical endpoints.

### 3.3 Mechanistic pathways: from neural circuits to cardiac outcomes

Post-stroke autonomic imbalance emerges from the coordinated collapse of hierarchical circuits within the CAN, representing a failure of the brain-heart axis (Valenza et al., 2025). To accurately navigate the underlying pathophysiology, it is essential to distinguish between the causal evidence established in experimental models and the clinical observations synthesized in Table 1. While human imaging indicates network-level correlations, animal lesion and stimulation studies provide the requisite causal proof for how central injury unmasks autonomic instability and triggers secondary cardiac injury.

**Table 1:** Multilevel evidence supporting central autonomic mechanisms in Stroke-Heart Syndrome.

Level of evidence	Experimental model/population	Nature of link/certainty	Key findings	Representative references
Molecular/cellular	Neuronal/glial cultures; rodent ischemia models	Causal (high)	Glutamate-mediated Ca^2+^ overload and neuronal apoptosis; NPY-driven sympathetic surge; NLRP3/IL-1β pathways acts as a downstream effector.	Cavallo et al. (2019), Dossat et al. (2024), Lee et al. (2022), Lin et al. (2024)
Animal (*in vivo*)	Rodent/primate models (lesion or stimulation)	Causal (high)	Direct insula–PVN–RVLM pathways modulate sympathetic/parasympathetic balance.	Butcher and Cechetto (1995), Chouchou et al. (2019), Marins et al. (2016, 2021)
Human neuroimaging/physiology	Stroke cohorts; fMRI, DTI, PET studies	Associative (moderate)	Insula-limbic network connectivity predicts HRV and myocardial biomarkers.	Scheitz et al. (2012), Scheitz et al. (2021), Templin et al. (2019), Winder et al. (2023)
Clinical correlates	Stroke patients (ECG/Troponin monitoring)	Applied (validated)	QTc dispersion, QT dispersion, and RMSSD reflect failed inhibitory CAN control.	Chang et al. (2020), Hanne et al. (2017), Siepmann et al. (2021)

The loss of top-down inhibitory control is the primary mechanistic driver of SHS. Evidence from rodent models demonstrates that inhibitory projections from the right insula to the PVN normally restrain hypothalamic sympathetic output; the loss of these projections leads to unchecked sympathetic surge (Butcher and Cechetto, 1995). In parallel, injury to the left insula disrupts the excitatory drive to NA and DVN, impairing baroreflex sensitivity and causing relative sympathetic overactivity via “vagal withdrawal” (Holtmann et al., 2022; Strain et al., 2024). These causal circuits explain why even small focal lesions can yield overlapping clinical phenotypes: the outcome depends not just on the locus of the infarct, but on the net imbalance of the inhibitory-excitatory loop within the distributed network.

At the molecular level, glutamate excitotoxicity serves as the pivotal intermediary mediating this central dysautonomia. Ischemic insults impair astrocytic glutamate transporters, leading to excessive synaptic glutamate accumulation. This overactivation of NMDA and AMPA receptors induces Ca^2+^ influx and nitrosative stress, particularly in the pathways connecting the insula and dorsomedial hypothalamus (Gill, 2022; Marins et al., 2021). This molecular cascade is further amplified by Neuropeptide Y (NPY) and immune-inflammatory axes. During sustained sympathetic activation, NPY co-released with norepinephrine enhances calcium loading in cardiomyocytes and sustains a self-perpetuating feedback loop through failure of the neuroprotective Y₂ feedback (McDowell et al., 2024; Shi et al., 2021). These central mechanisms act in concert with peripheral cascades—specifically the NLRP3/IL-1β and catecholamine-dependent bone marrow activation—to promote myocardial fibrosis and diastolic dysfunction (Lin et al., 2024; Simats et al., 2024).

Functionally, these multilevel perturbations integrate into the characteristic electrocardiographic signatures observed in SHS. The sympathetic-vagal imbalance—driven by central circuit disintegration—increases the regional dispersion of action potential duration, prolonged QTc, and QT dispersion (Fink et al., 2011; Silvani et al., 2016; Zandstra et al., 2021). These repolarization labilities are the distal readouts of failed central inhibitory neurocircuitry, bridging the gap between molecular triggers and cardiac manifestations. By identifying these conserved, network-wide responses to focal brain injury, SHS can be transitioned from a cluster of disparate cardiac symptoms into a unified, neuro-centric disorder.

## 4 The limbic system and distributed cortical networks: integrative hub for autonomic, emotional, sensory–motor, and cognitive processing

The limbic system constitutes a complex and hierarchically organized network comprising the amygdala, cingulate cortex, hypothalamus, hippocampus, anterior thalamic nuclei, and select prefrontal regions. These interconnected structures govern autonomic and endocrine regulation, emotional and motivational processing, memory formation, and homeostatic balance (Bo et al., 2024; Hu et al., 2021; Ruiz Vargas et al., 2016; Šimić et al., 2021). Disruption of inhibitory feedback circuits within this system amplifies excitatory autonomic output and emotional reactivity (Mujica-Parodi et al., 2009). Importantly, the term “limbic” is used here heuristically. In humans, autonomic regulation arises from dynamic, task-dependent coupling between limbic hubs and distributed cortical networks, including the sensorimotor, salience, default-mode, and executive control systems within the broader CAN (Templin et al., 2019). During stroke and autonomic challenges, these hubs co-activate with premotor/motor, somatosensory, and lateral temporal cortices, forming a distributed autonomic network rather than a purely emotion-centered system (Beissner et al., 2013; Chouchou et al., 2025; Critchley and Harrison, 2013; Gianaros and Jennings, 2018). Below we elaborate mechanistic routes through which the amygdala and hippocampus interact with the hypothalamus, brainstem, and insula to shape cardiovascular control in SHS. Below, we outline key circuits—particularly those involving the amygdala, hippocampus, and ACC—that link limbic structures to hypothalamic, brainstem, and insular regions in regulating cardiovascular function in SHS.

Functional neuroimaging reveals that patients with TTS display increased resting connectivity within a network comprising the anterior insula, ACC, hippocampus, and parahippocampal gyrus—regions engaged in emotional and autonomic integration (Silva et al., 2019). During sympathetic arousal, enhanced coupling among the amygdala, putamen, right insula, and cerebellum mirrors that observed in SHS, underscoring shared limbic-autonomic dysregulation (Marle et al., 2010). The insula and ACC serve as core integration nodes for ascending interoceptive input and descending autonomic modulation (Craig, 2002; Yoshimoto et al., 2024). Population-based imaging further links elevated resting amygdalar metabolic activity to increased risk of major cardiovascular events, mediated through bone-marrow activation and arterial inflammation, delineating a limbic–immune–cardiovascular axis central to SHS pathogenesis (Tawakol et al., 2017).

The ACC bridges emotional and executive domains with autonomic regulation through extensive reciprocal connections to limbic and brainstem nuclei (Seamans and Floresco, 2022). It exerts bidirectional control over sympathetic and parasympathetic outflow, influencing heart rate, blood pressure, and HRV (Critchley et al., 2003; Gillies et al., 2019; Guo et al., 2016; Oane et al., 2020). Anatomically, its efferent projections target the amygdala, hypothalamus, and medullary autonomic centers (Koga et al., 2020; McDonald et al., 1999; Van Eden and Buijs, 2000), allowing precise modulation of visceral states. The affective subregions of the ACC (Brodmann areas 25/33) influence autonomic balance via outputs to the hypothalamus and insula (Devinsky et al., 1995). Signals can propagate through the hippocampus–amygdala–hypothalamus pathway to the brainstem reticular formation, modulating HR and BP (Chen P. C. et al., 2022). In parallel, ACC projections to the ventromedial thalamic nucleus activate dorsomedial hypothalamic neurons projecting to the vagal nucleus, thereby modulating parasympathetic output (Yoshimoto et al., 2024). Task-based imaging confirms that ACC activity covaries with HR and HRV during cognitive, pain, and interoceptive tasks, linking executive load to autonomic tone (Beissner et al., 2013; Gianaros and Wager, 2015; Shoemaker, 2022).

The central nucleus of the amygdala (CeA) connects densely with the hypothalamus, NTS, and brainstem reticular formation (Ponserre et al., 2020). Under stress or fear, the CeA triggers sympathetic excitation (“fight-or-flight” response) while suppressing vagal activity (Dimsdale and Moss, 1980; Pelliccia et al., 2017). Through direct projections to the NTS and dorsal vagal complex, the CeA participates in cardiovascular and visceral modulation (van der Kooy et al., 1984). The basolateral amygdala (BLA) receives polymodal cortical inputs—particularly from the insula and prefrontal cortex—and transmits contextual and sensory information to the CeA and the bed nucleus of the stria terminalis (BNST) (Ishikawa et al., 2020; Jhang et al., 2018). CeA outputs to the hypothalamus, parabrachial nucleus, and RVLM enable rapid adjustments of HR and BP during threat. Experimental microstimulation and pharmacological modulation of BLA/CeA in animal models bidirectionally alter vasomotor tone and cardiovagal outflow, providing a mechanistic substrate for stress-induced arrhythmias and QT variability in SHS (Aukema et al., 2024). BNST projections to the PVN maintain prolonged autonomic arousal, explaining the persistence of dysautonomia following stroke-related limbic disinhibition (Bingham et al., 2011; Dabrowska et al., 2013).

The prefrontal cortex modulates autonomic function by inhibiting amygdalar output and enhancing vagal tone (Alexandra Kredlow et al., 2022; Skog et al., 2024). The hypothalamus acts as the central integrator, coordinating input from the ACC, amygdala, and hippocampus to regulate sympathetic–parasympathetic balance via the NTS and ventrolateral medulla (Carrillo-Franco et al., 2024; Henderson and Macefield, 2021). The hippocampus, through its interaction with the HPA axis, restrains PVN activity and sympathetic drive while supporting vagal output (Radley and Sawchenko, 2015; Zhang X. F. et al., 2024). Experimental stimulation of hippocampal/subicular pathways elicits bradycardia and hypotension, whereas hippocampal lesions enhance glucocorticoid responses, tachycardia, and HRV reduction—mirroring stress-sensitive aspects of SHS (Ruit and Neafsey, 1988; Scopinho et al., 2013). Through fornical and septo-hypothalamic relays, hippocampal output integrates contextual memory with interoceptive and executive signals, adjusting baroreflex set-points under stress and recovery (Bär et al., 2016; Shoemaker and Goswami, 2015).

Beyond emotion-centric frameworks, the limbic system operates in concert with sensorimotor and cognitive control networks to tune autonomic activity during movement, sensation, and cognition (Luque-Casado et al., 2016; Shoemaker, 2022; Tan et al., 2018). Motor preparation, isometric effort, and somatosensory stimulation co-activate the anterior insula, ACC, and premotor–sensorimotor cortices, producing predictable HR, BP, and HRV responses (Duque and Ivry, 2009; Seifert et al., 2013; Wong et al., 2007). Pain and interoceptive attention couple insula–cingulate activation to transient sympathetic bursts, while cognitive tasks such as mental arithmetic and inhibitory control recruit dorsolateral prefrontal cortex (DLPFC)–ACC circuits that adjust autonomic tone in a load-dependent fashion (Coelho et al., 2025; Riganello et al., 2023). Thus, post-stroke dysautonomia reflects disruption of a prefronto-limbic–sensorimotor macro-network rather than an isolated limbic subsystem. Nonhuman primate tracing confirms this distributed organization, revealing direct cortical projections from motor, premotor, cingulate, and medial prefrontal areas to autonomic effectors such as the adrenal medulla (Dum et al., 2016).

Collectively, these findings highlight the limbic system as a dynamic integrator within a distributed cortical–subcortical hierarchy. Structural or functional lesions—particularly those involving insula–amygdala or hippocampal–hypothalamic connections—disturb autonomic homeostasis and precipitate SHS. Recognizing this cross-network integration prevents an overly narrow emotional interpretation of brain–autonomic coupling and aligns mechanistic frameworks with observed clinical and physiological phenotypes in SHS. An integrated summary of supporting evidence is provided in Table 2.

**Table 2:** Central autonomic network contributions to autonomic regulation in Stroke-Heart Syndrome.

Structure/network	Functional role in autonomic regulation	Evidence type/model	Key findings	Representative references
Amygdala	Drives sympathetic excitation and fear-related responses; integrates visceral and emotional signals.	Animal models; human fMRI	CeA–NTS projections mediate HR/BP control; increased amygdalar metabolism predicts post-stroke cardiac events.	Pelliccia et al. (2017), Ponserre et al. (2020), Tawakol et al. (2017)
Hippocampus	Inhibits HPA axis and facilitates vagal tone; modulates stress reactivity.	Rodent electrophysiology; clinical imaging	Ventral hippocampal activation → bradycardia; lesions → tachycardia + ↑corticosterone; restrains PVN output.	Cole et al. (2022), Scopinho et al. (2013)
Anterior cingulate cortex	Integrates emotion, cognition, and autonomic output; coordinates sympathetic–parasympathetic balance.	Human fMRI/ direct stimulation	ACC activity correlates with HRV; projects to hypothalamus and brainstem; ACC–DMH pathway regulates vagal tone.	Critchley et al. (2003), Fillinger et al. (2018), Yoshimoto et al. (2024)
Prefrontal cortex	Provides top-down inhibitory control over amygdala and hypothalamus; enhances vagal function.	Human neuroimaging; nVNS studies	PFC–amygdala coupling predicts HRV; DLPFC stimulation ↑parasympathetic tone.	Alexandra Kredlow et al. (2022), Makovac et al. (2017), Skog et al. (2024)
Hypothalamus	Central hub linking limbic inputs to autonomic and endocrine output.	Rodent + human PET data	PVN integrates limbic inputs to drive sympathetic–HPA co-activation; lesions → autonomic collapse.	Carrillo-Franco et al. (2024), Marins et al. (2021)
Insula	Converts interoceptive and emotional signals into autonomic commands; key interface of CAN.	Lesion mapping; fMRI	Right insula → sympathetic activation; left insula → vagal predominance; disrupted insula–amygdala coupling → arrhythmia.	Keller et al. (2020), Sanchez-Larsen et al. (2024)
Sensorimotor and cognitive networks	Integrate movement, sensation, and executive load with autonomic output.	Human task-fMRI, EEG	SMA/M1/S1 and DLPFC–ACC coupling modulates HR/BP/HRV during motor and cognitive tasks.	Coelho et al. (2025), Shoemaker (2022)

## 5 Clinical implications and future directions

### 5.1 Neuroimaging and risk stratification: establishing the parsimonious core dataset

Recent advances in neuroimaging have substantially refined our understanding of CAN injury and its relevance to SHS. Advanced modalities like fMRI, DTI, and PET now enable high-resolution detection of structural and functional abnormalities in key nodes (insula, ACC, amygdala) (Catalano et al., 2025; Ruiz Vargas et al., 2016). As evidenced by multivariate lesion mapping, acute infarction of the right insula and adjacent limbic regions strongly correlates with reduced LVEF (Winder et al., 2023), reinforcing the central role of autonomic–limbic integration. Critically, the emerging integration of AI accelerates the translation of these research insights by enabling the automated, high-throughput extraction of standardized endpoints from routine diagnostics (Braillon et al., 2023).

To facilitate clinical translation, it is essential to distinguish research-grade biomarkers from near-term clinical tools. While PET markers of glial activation and whole-brain connectome analysis are essential for understanding pathophysiology (Hermanns et al., 2022), they currently remain Research-Grade due to high resource intensity, the lack of universal standardization, and the logistical challenges of obtaining high-quality, motion-free sequences in acute, clinically unstable stroke patients. In contrast, a pragmatic near-term approach can leverage routinely available data to identify patients at heightened cardiac risk.

Based on current evidence, intensified cardiac monitoring should be prioritized for patients with moderate-to-severe stroke, early hs-cTn elevation, or neuroimaging evidence of CAN involvement. We propose a parsimonious core dataset suitable for multicentre implementation, comprising:

Near-term (multicentre feasible core dataset)

- Demographics & stroke descriptors: age, sex, NIHSS, stroke subtype, lesion laterality (insula/limbic/brainstem).
- Continuous ECG telemetry: 72 h (start at admission).
- Standardized ECG endpoints: automated QTc (Bazett), QTc dispersion, and beat-to-beat repolarization lability.
- HRV indices (time-domain): RMSSD, SDNN (report method & epoch length).
- hs-cTn schedule: baseline, 6 h, 24 h, 48 h, 72 h (report assay and ULN multiples).
- Bedside transthoracic echocardiography: trigger if new ECG repolarization change or hs-cTn rise >20% or clinical instability; report LVEF and RWMA pattern.
- Core outcomes: new clinically significant arrhythmia, in-hospital MACE, 30-day mortality.

Research-grade (optional, specialized)

- DTI/tractography connectomics, PET glial markers (TSPO), high-field fMRI connectome metrics, centralized AI lesion-topology pipelines.

By standardizing these minimalist yet rigorous endpoints, the field can shift from descriptive observation toward a standardized predictive model.

### 5.2 Targeted interventions: mechanisms and implementation tiers

Beyond risk identification, therapeutic strategies aim to restore autonomic balance and mitigate downstream cardiac injury. Interventions such as HRV biofeedback, non-invasive vagus nerve stimulation (nVNS), and non-invasive brain stimulation (NIBS) target different levels of the CAN–autonomic axis. Experimental and early clinical studies suggest that these approaches can modulate cortical–subcortical circuits, enhance vagal tone, and attenuate sympathetic overactivity (Jiao et al., 2024; Machetanz et al., 2021; Yoo et al., 2023).

Despite this mechanistic promise, the translational pathway faces pragmatic constraints. Effective implementation often requires preserved patient cognition (particularly for active biofeedback tasks), and there remains a paucity of long-term trial data confirming that acute autonomic modulation translates into reduced post-stroke mortality. To address these gaps and support realistic uptake, we propose a feasibility hierarchy: Tier 1 (near-term applicability) includes low-cost, scalable interventions such as HRV biofeedback and nVNS, monitored using established physiological endpoints (e.g., HRV indices and arrhythmia burden). Tier 2 (research frontier) encompasses NIBS and closed-loop neuromodulation strategies integrating advanced neuroimaging or biomarker feedback. Broad clinical uptake will require prospective validation using standardized cardiac endpoints and harmonized monitoring protocols across centers.

## 6 Conclusion

Stroke-Heart Syndrome represents a disorder of neuro-cardiac dysregulation arising from the structural and functional decoupling of the insula–limbic hub within the central autonomic network. The ensuing loss of top-down inhibitory control unleashes a cascade of glutamate excitotoxicity, neuroinflammation, and sympathetic hyperactivation, culminating in direct myocardial injury, impaired contractility, and malignant arrhythmias.

Multimodal neuroimaging now enables *in vivo* visualization of this brain–heart disconnection, offering quantitative biomarkers to identify high-risk patients and guide the development of neurocentric therapeutic strategies. Interventions such as heart-rate-variability biofeedback and non-invasive neuromodulation show growing promise in restoring autonomic homeostasis by re-engaging these disrupted cortical–subcortical circuits. Ultimately, translating mechanistic insights into clinical benefit requires a multidimensional paradigm shift: combining neuroimaging-based risk stratification with targeted neuromodulation and molecular interventions. Such an approach holds the potential to fundamentally mitigate the burden of post-stroke cardiac morbidity and mortality, transforming SHS management from reactive to preventive, from peripheral symptom control to central network restoration.
